# Supplementary material for: Polydiacetylene-based ultrastrong bioorthogonal Raman probes for targeted live-cell Raman imaging
Source: Nat Commun. 2020 Jan 3;11:81. doi: 10.1038/s41467-019-13784-0 (PMC6941979; doi:10.1038/s41467-019-13784-0)
Supplement: Supplementary file 1 — Supplementary Information [file 41467_2019_13784_MOESM1_ESM.pdf]

Supplementary Information：

Polydiacetylene-Based Ultrastrong Bioorthogonal Raman Probes for Targeted Live-Cell Raman Imaging

Tian et al.

Table of contents

Supplementary Methods S3
Materials S3
Synthesis Methods S3
Instruments and Characterization Methods S4
Cell Study Methods S5
Imaging Methods S6
Supplementary Figure 1 .... S7
Supplementary Figure 2 .... S8
Supplementary Figure 3 .... S9
Supplementary Figure 4 .... S10
Supplementary Figure 5 .... S11
Supplementary Table 1 .... S12
Supplementary Figure 6 .... S13
Supplementary Figure 7 .... S14
Supplementary Figure 8 .... S15
Supplementary Table 2 .... S16
Supplementary Figure 9 .... S17
Supplementary Figure 10 .... S18
Supplementary Figure 11 .... S19
Supplementary Figure 12 .... S20
Supplementary Figure 13 .... S21
Supplementary Figure 14 .... S22
Supplementary Figure 15 .... S23
Supplementary Figure 16 .... S24
Supplementary Figure 17 .... S25
Supplementary Figure 18 .... S26
Supplementary Reference S26

Supplementary Methods

1.Materials

4-Pentyn-1-ol and 5-ethynyl-2'-deoxyuridine were purchased from Tokyo Chemical Industry. 1,4-Diphenylbutadiyne, 4-Pyridinemethaneamine, N-(3-Dimethylaminopropyl)-N'-ethylcarbodiimide hydrochloride (EDCI), Cuprous chloride (CuCl), N,N-Diethylethylenediamine (DEA) were purchased from Energy Chemical. Mal-PEG_{2k}-NH_2 was purchased from HuaTeng Pharma. Diethyl oxalate, Chromium oxide (Cr_2O_3), N,N,N',N'-Tetramethylethylenediamine (TMEDA), N-Hydroxysuccinimide (NHS) were purchased from Sinopharm Chemical Reagent Co. Ltd. N-Hydroxysulfosuccinimide sodium salt (NHSS) were purchased from Meryer (Shanghai) Chemical Technology Co. Ltd. Solid state synthesis of the targeting peptide (CGKRK and TAT-SH) were conducted by GL Biochem as customized products with 95% purity. Unless otherwise noted all reagents with analytical purity were used as received. All other reagents and solvents were purchased from Sinopharm Chemical Reagent Co. Ltd. and used as received.

2. Synthesis Methods

Deca-4,6-diynedioic acid (DDA): Deca-4,6-diyne-1,10-diol (0.83 g, 5 mmol) is dissolved in 100 mL cold acetone in an ice-water bath. Freshly made Jones reagent (30 mL) is added dropwise to the deca-4,6-diyne-1,10-diol solution under vigorous stirring during a time of 30 minutes. After reacting at 0 ºC for overnight, the mixture is quenched by isopropyl alcohol and filtered to remove the solid. Remove the solvent under rotation vacuum, and dilute the remaining residue with 100 mL of water. The pH

of the aqueous solution is adjusted to 12 with NaOH, and then washed with ethyl acetate (30 mL×3) before acidified with hydrochloric acid. The acidic water phase is extracted with ethyl acetate (50 mL×5) and the combined organic phase is washed with dilute hydrochloric acid (30 mL×3), brine (30 mL×3) and then dried over sodium sulfate. After evaporation in vacuum, the resulting crude product is loaded on silica gel column. Column chromatography (ethyl acetate) yields a white solid (0.68 g, yield: 70%). ^{1}H-NMR (400 MHz, methanol-d_4) chemical shift (ppm) 2.49 (t, J = 7.2 Hz, 4H), 2.39 (t, J = 7.2 Hz, 4H). ^{13}C-NMR (100 MHz, methanol-d_4) chemical shift (ppm) 179.8, 77.5, 64.7, 49.0, 34.7. HR-MS (m/z): $[M+Na]^+$ Calculated for $C_{10}H_{10}O_4Na^+$, 217.04713; found: 217.04744.

3. Instruments and Characterization Methods

Gel permeation chromatography (GPC): GPC is conducted with an Agilent 1100/1200 series liquid phase chromatography system equipped with G1315B DAD UV-Vis Abs detector recording at the wavelength of 460 nm. PL aquagel-OH MIXED 7.5 mm×300,8 μm column is used for the separation. A mixed buffer of 100 mM NO_3^- and 10 mM PO_4^{3-} with Na^+ as counter-ion (pH 9, adjusted with NaOH) is used as eluent with a flow rate of 1.0 mL min^{-1}. Concentration of the samples is fixed at 0.2% (w/w). A series of low polydispersity dextran standards were employed for calibration.

Mass spectrometry: Electro-spray ionization mass spectrometry (ESI-MS) experiment was performed on an Agilent LC/MSD Trap XCT Mass Spectrometer equipped with an electrospray interface working in negative ion mode.

UV-Vis absorption spectroscopy: All UV-Vis spectra were acquired on a TU-

1810DSPC UV/Vis spectrophotometer (Puxi. General Instrumental Company, China). 2.5 mL of liquid sample was charged into a quartz cuvette with 1 cm optical path and scanned within wavelength range from 800 nm to 300 nm.

Fluorescence spectroscopy: Fluorescence spectra were recorded on a Hitachi F-4600 Fluorescence Spectrophotometer. To match the excitation condition in confocal microscopy, the fluorescence spectra acquisition for PDDA and its derivatives were excited at 488 nm. The slit widths were both set at 5 nm for excitation and emission. PMT voltage were set at 700V.

Fourier transform infrared spectroscopy： FTIR spectra were collected using a Bruker Alpha FTIR spectrometer equipped with a diamond ATR window. Data were collected at ambient temperature from 4000 cm^{-1} to 500 cm^{-1}

4. Cell Study Methods

MTT test: Freshly prepared **P2**, **P3**, and **P4** solutions with desired concentrations in DMEM containing 10% (v/v) fetal bovine serum (FBS) and antibiotics (penicillin/streptomycin) were added to the cells in a 96-well plate. To avoid possible edge effect, only the central 6×10 wells were used. The PDDA-based probes containing culture medium was removed after 12, 24, or 48 h. 20 μL of 5 mg mL^{-1} MTT solution in PBS was added and incubated for additional 4 h. After the removal of the culture medium, the cells were washed with 1× PBS for 3 times before 200 μL DMSO was added to each well and shaken for 15min. The OD 570 nm of the samples were measured with a Thermo-fisher Varioskan LUX micro-plate reader. 6 replicates were conducted for each sample.

5. Imaging Methods

Fluorescence confocal microscopy: Confocal imaging was performed using Olympus FV3000 microscope with 40× objective lens. An optical zoom of ×5 was applied. The PDDA fluorescence was excited with 488 nm laser and the emission collection range was set from 500 nm to 550 nm. The fluorescence staining for different organelles were conducted with Lyso-tracker Red, Mito-tracker Deep Red and DAPI. The fluorescence for Lyso-tracker Red, Mito-tracker Deep Red were excited with 561 nm laser to avoid possible interference from PDDA. The emission collection range was set from 600 nm to 700 nm. The fluorescence for DAPI was excited with 405 nm laser to avoid possible interference from PDDA. The emission collection range was set from 420 nm to 470 nm.

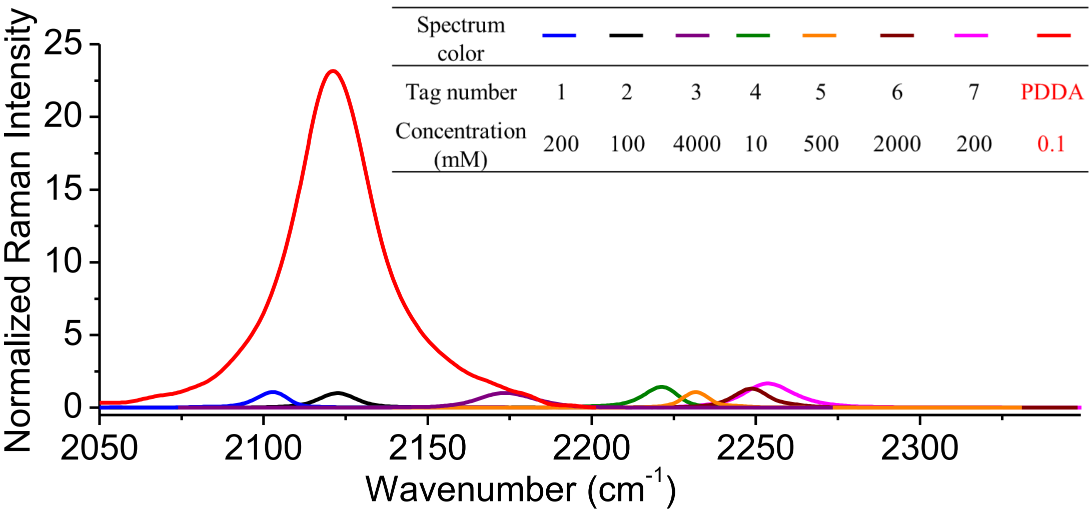

Tag number	Compound Name	Chemical Structure	Raman Active bond	Raman Shift/cm^{-1}
1	Ethynylbenzene	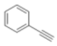	C≡C	2103
2	EdU	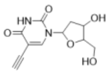	C≡C	2122
3	Diphenyl phosphorazidate	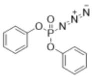	N_3	2173
4	DPY	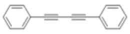	C≡C	2219
5	Benzonitrile	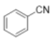	C≡N	2232
6	5-Bromopentanenitrile	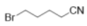	C≡N	2249
7	DDA	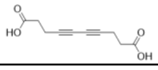	C≡C	2254

Supplementary Figure 1. Overlaid Raman spectra of individual DMSO solution of PDDA and a series of representative vibrational tags, including 1. Ethynylbenzene; 2. EdU; 3. Diphenyl phosphorazidate; 4. Diphenylbutadiyne (DPY); 5. Benzonitrile; 6. 5-Bromopentanenitrile; 7. Deca-4,6-diynedioic acid (DDA). The inset table lists the concentration of Raman-active bonds (C≡C, C≡N, or azide) in each solution. The normalization is based on the absolute Raman intensity of each spectrum. The table at the bottom contains detailed information of the Raman tags.

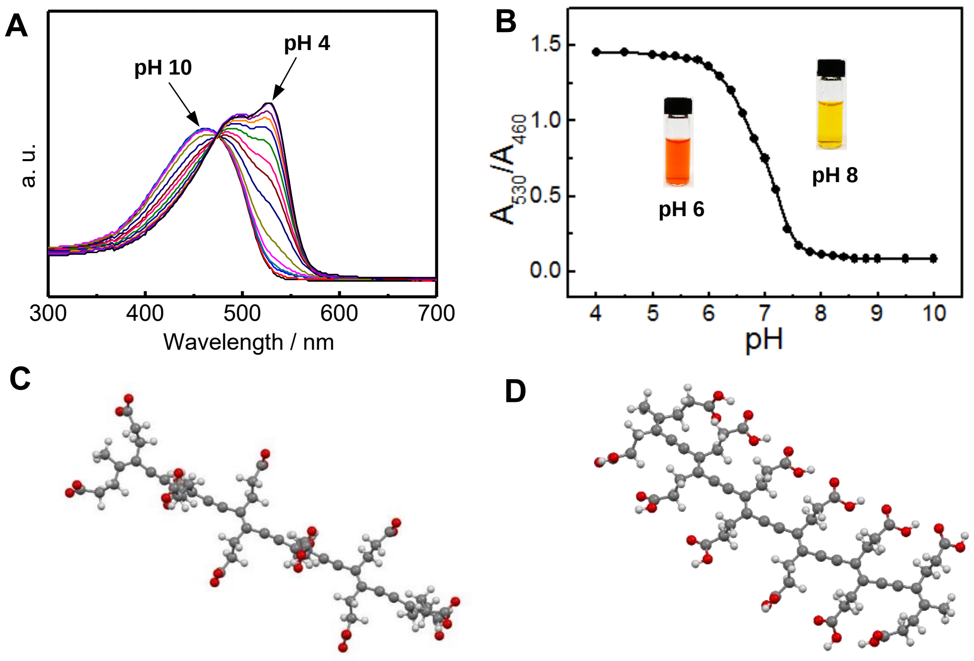

Supplementary Figure 2. Absorption spectra and density functional theory calculation for PDDA. A) UV/Vis absorption spectra of a PDDA water solution at different pH values in the range of pH 4 to pH 10. B) The ratio of the solution absorption at 530 nm and at 460 nm as a function of pH value. The insets show the color change of the solution. C-D) Density functional theory (DFT) calculation with b3lyp/6-31g(d) method of the conformations of a PDDA fragment (6 repeating units) with its carboxyl group in deprotonated (C) and protonated (D) forms.

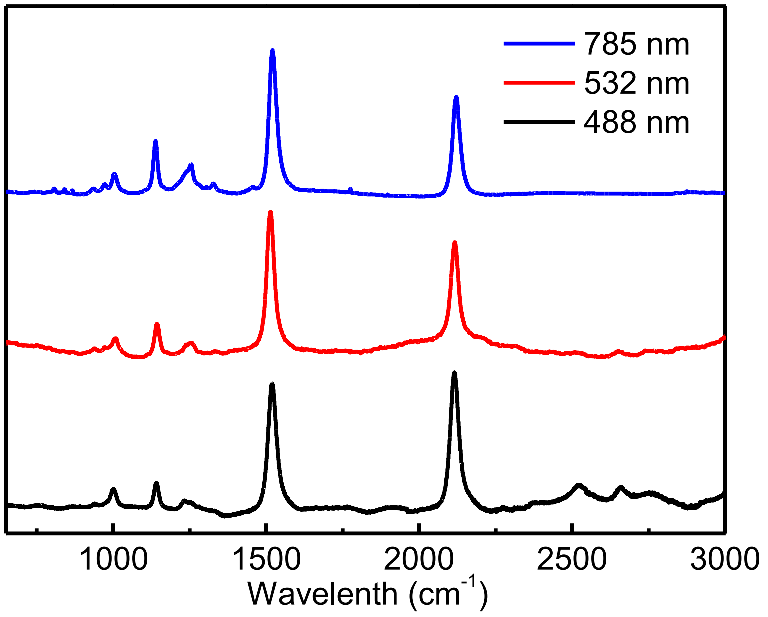

Supplementary Figure 3. Spontaneous Raman spectra of a PDDA DMSO solution under excitation at different wavelengths.

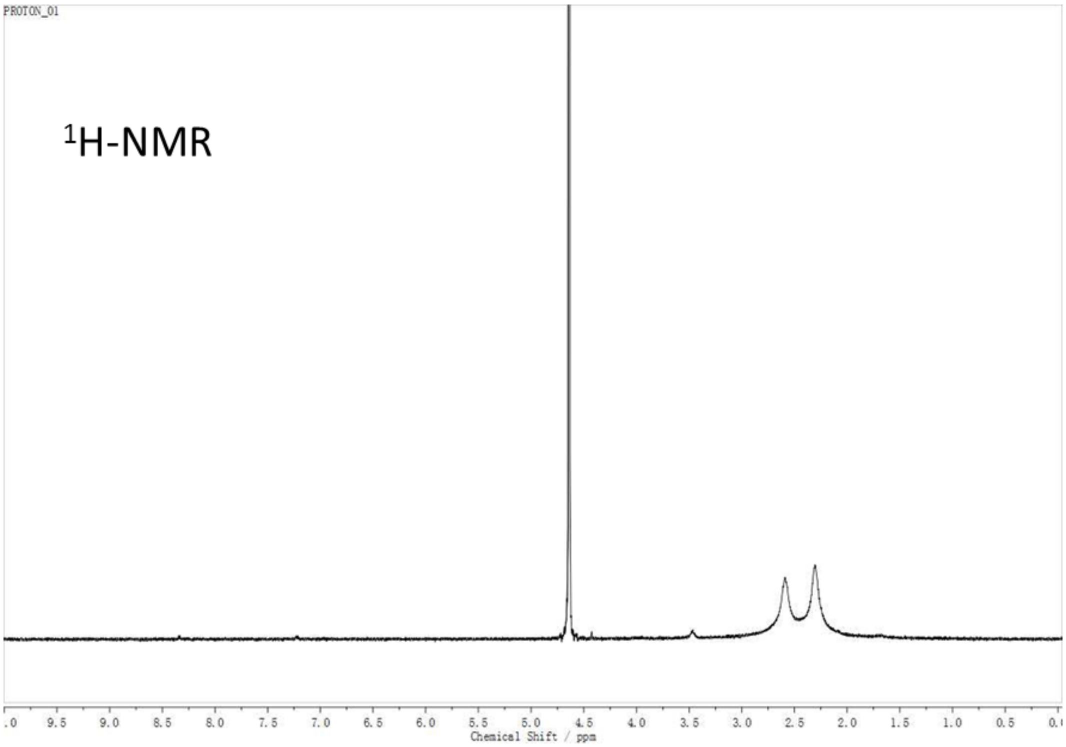

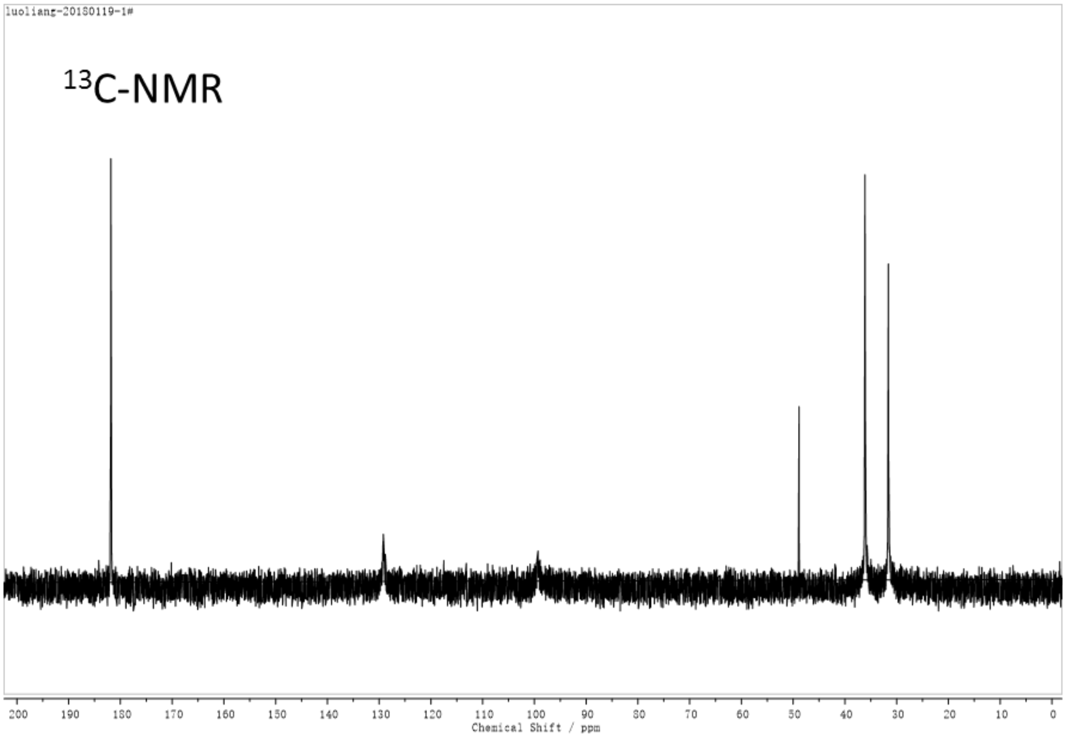

Supplementary Figure 4. ^{1}H-NMR and ^{13}C-NMR of the PDDA polymer after host removal in basic D_2O. The sample in D_2O is basified with sodium carbonate to achieve a satisfactory polymer concentration.

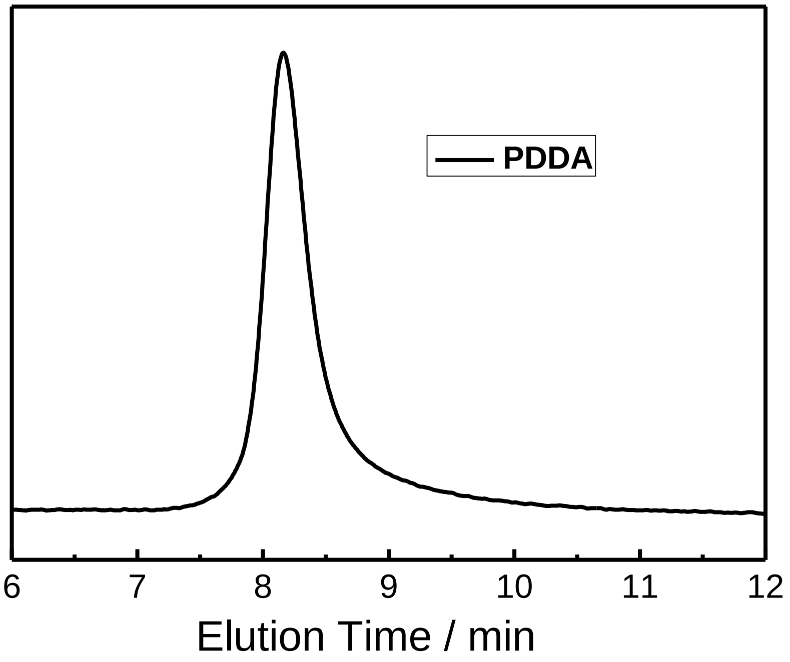

Supplementary Figure 5. Gel Permeation Chromatography (GPC) of PDDA with basic aqueous medium as the mobile phase. A mixed buffer of 100 mM NO_3^- and 10 mM PO_4^{3-} with Na^+ as counter-ion (pH 9, adjusted with NaOH) is used as eluent with a flow rate of 1.0 mL min^{-1}. Concentration of the samples is fixed at 0.2% (w/w).

Supplementary Table 1. A comparison of PDDA with literature reported polydiacetylenes.

sample	M_n (g mol^{-1})	M_w (g mol^{-1})	PDI	Reference
PDDA	2.4×10^4	3.3×10^4	1.45	This work
P2c[a]	2×10^6	9×10^6	4.5	Ref.[1]
P3BCMU	1.3×10^6	3.9×10^6	3.0	Ref.[2]
P4BCMU	1.6×10^5	5.5×10^5	3.4	Ref.[3]
P4BCMU	n.a.	n.a.	5.0	Ref.[4]

a. The values of this polymer may suggest (partial) aggregation and shear-induced degradation of the polymer on the column, as indicated by the authors.

P2c

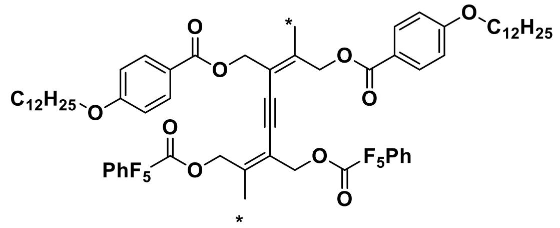

P3BCMU

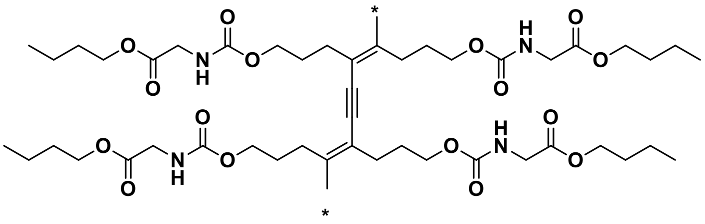

P4BCMU

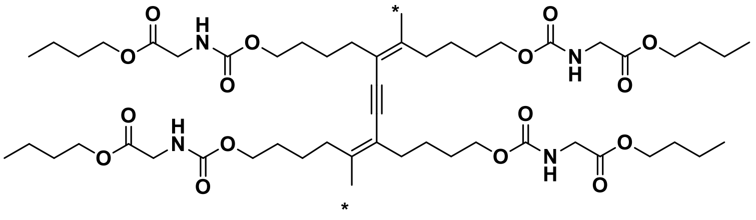

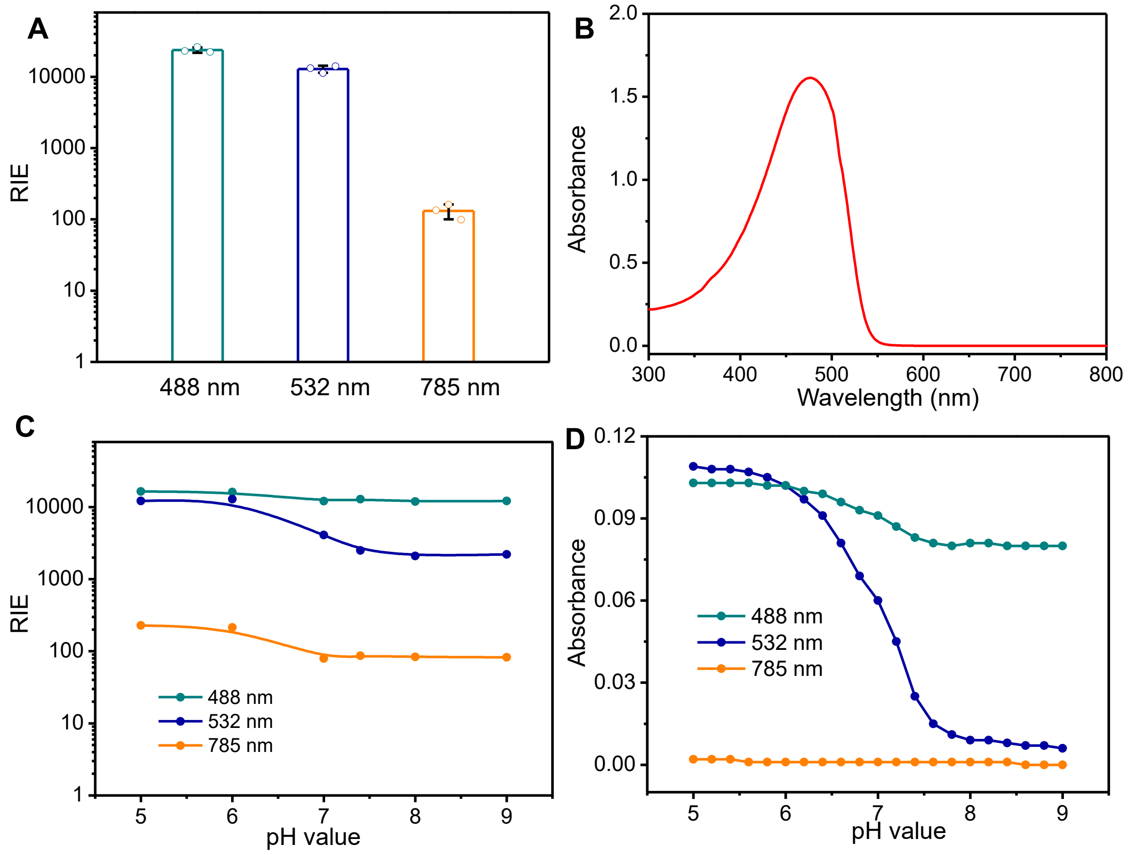

Supplementary Figure 6. Comparison of RIE values of PDDA at different excitation wavelenths. A) RIE values of PDDA in DMSO measured with different Raman excitation wavelengths (488 nm, 532 nm, and 785 nm). Data is represented as mean ± standard deviation (n=3). B) UV/Vis absorption spectrum of a DMSO solution of PDDA. C) pH-dependent RIE values of PDDA measured at different Raman excitation wavelengths (488 nm, 532 nm, and 785 nm). D) pH dependent absorption spectra of PDDA in aqueous medium at different wavelengths (488 nm, 532 nm, and 785 nm).

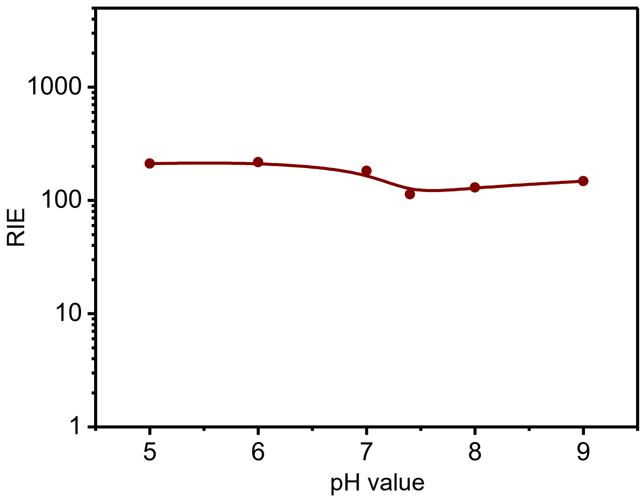

Supplementary Figure 7. RIE values of PDDA as a function of pH values measured by SRS (excitation: 853 nm; pump: 1040 nm).

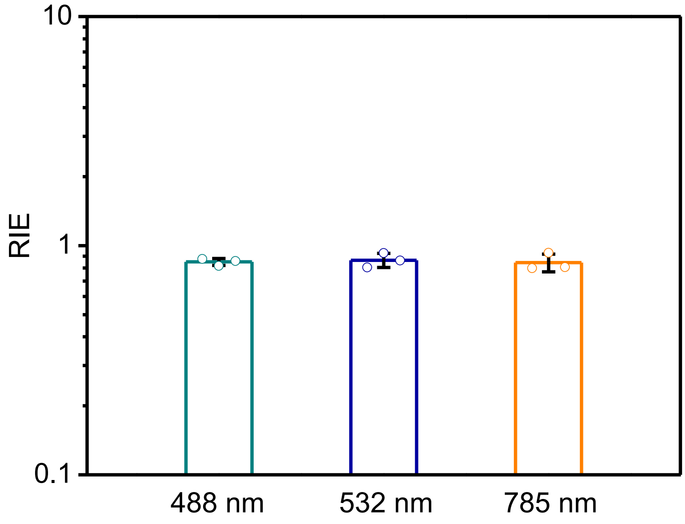

Supplementary Figure 8. RIE values of DDA in DMSO measured with different Raman excitation wavelengths (488 nm, 532 nm, and 785 nm). Data is represented as mean ± standard deviation (n=3).

Supplementary Table 2. Detailed information of each point in Figure 5.[a]

Acetylenes[b]	RIE	CRIE	Raman shift (cm^{-1})	Diynes[c]	RIE	CRIE	Raman shift (cm^{-1})
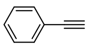	0.49	0.49	2102	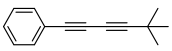	5.3	2.65	2237
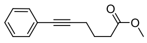	0.63	0.63	2228	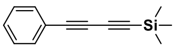	7.8	3.9	2207
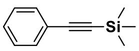	0.93	0.93	2157	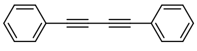	27	13.5	2219
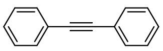	4.2	4.2	2219	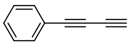	4.8	2.4	2208
				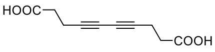	1.7	0.85	2254

PPEs[d]	RIE	CRIE	Raman shift (cm^{-1})	Polyynes[e]	RIE	CRIE	Raman shift (cm^{-1})
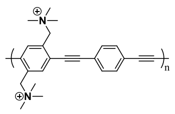	n.a.[d]	1.75	2200	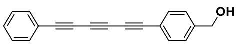	63	21	2185
				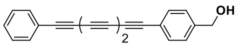	144	36	2140
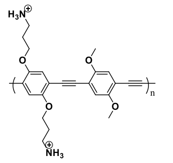	n.a.[d]	8	2200	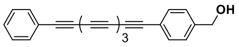	275	55	2100
				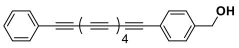	450	75	2060

a. RIE: molecular relative intensity versus EdU; CRIE: C≡C bond-normalized RIE
b. The data were collected from the reference 5;
c. The data were collected from the reference 6. The molecular weight information is not available in the reference;
d. The data were collected from the reference 7.

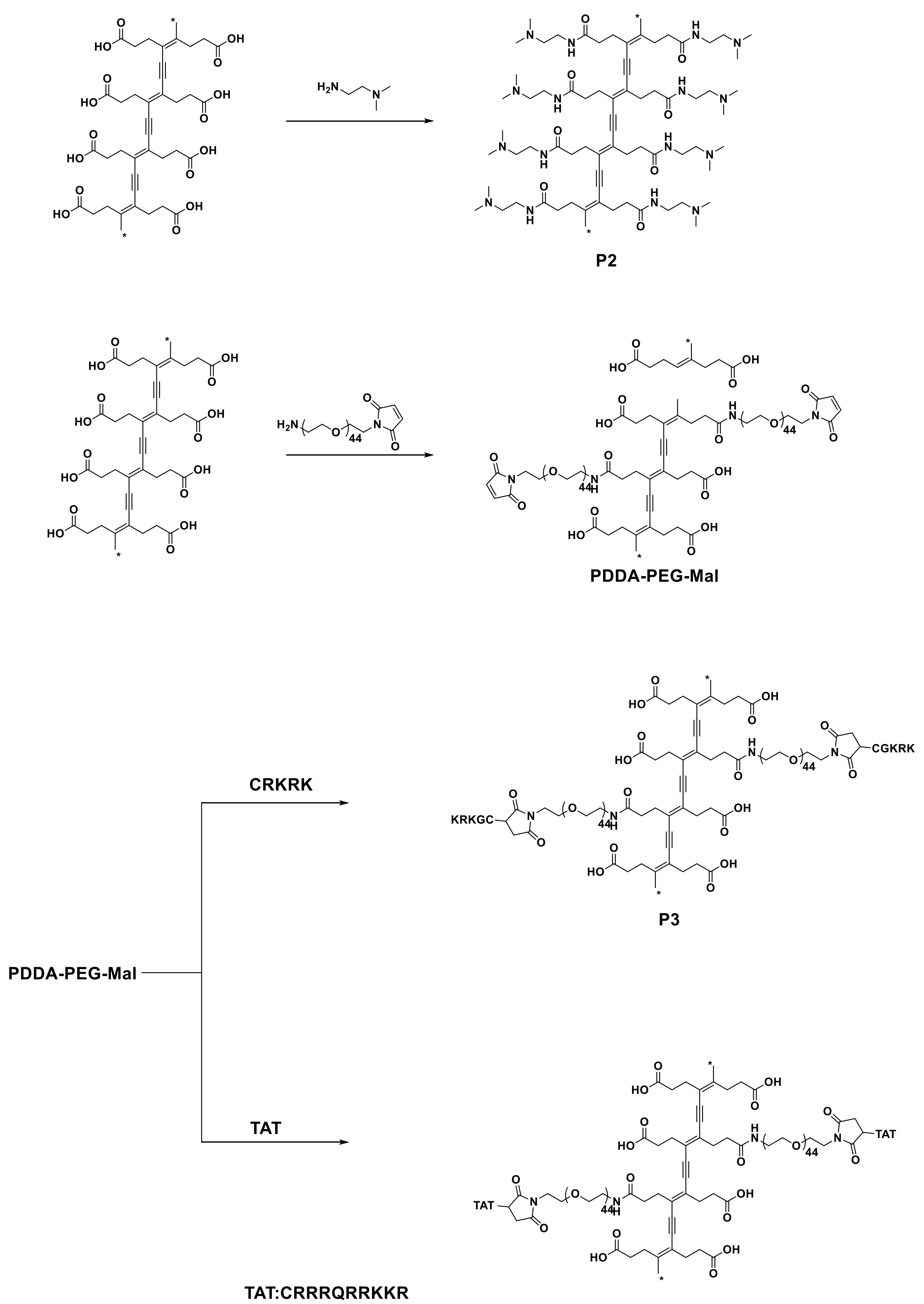

Supplementary Figure 9. Synthetic routes for the preparation of targeting group functionalized PDDA derivatives **P2**, **P3**, and **P4**.

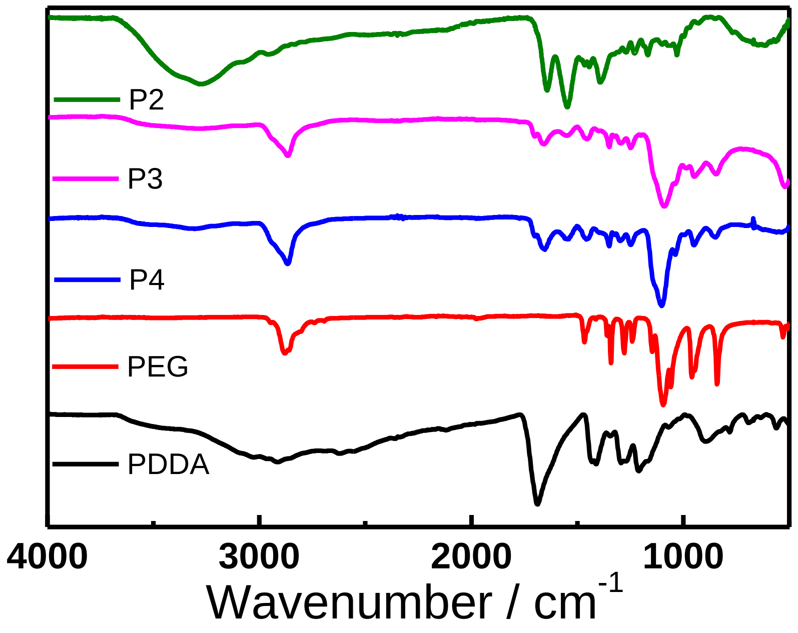

Supplementary Figure 10. FTIR spectra of the PDDA and targeting group functionalized derivatives.

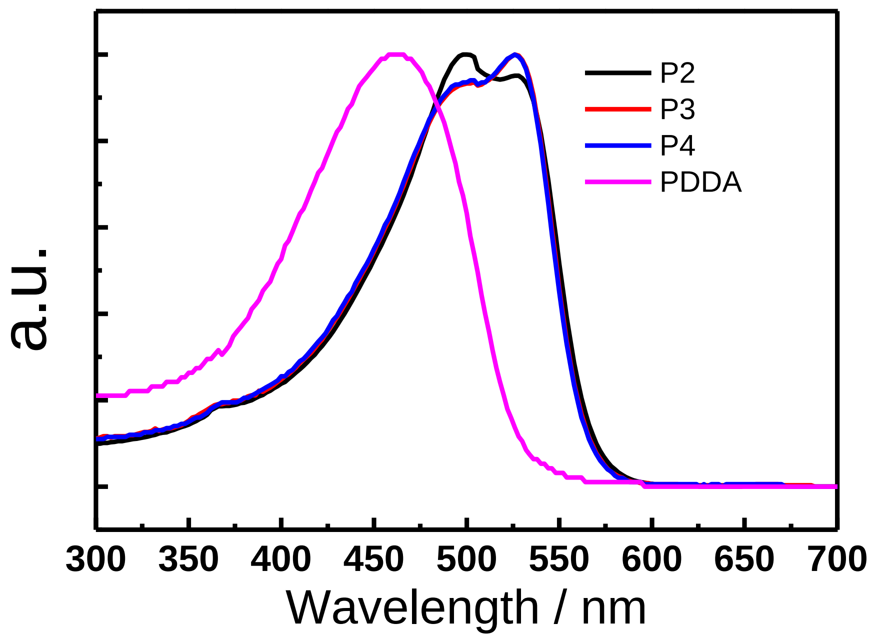

Supplementary Figure 11. UV-Vis absorption spectra of the targeting group functionalized PDDA derivatives.

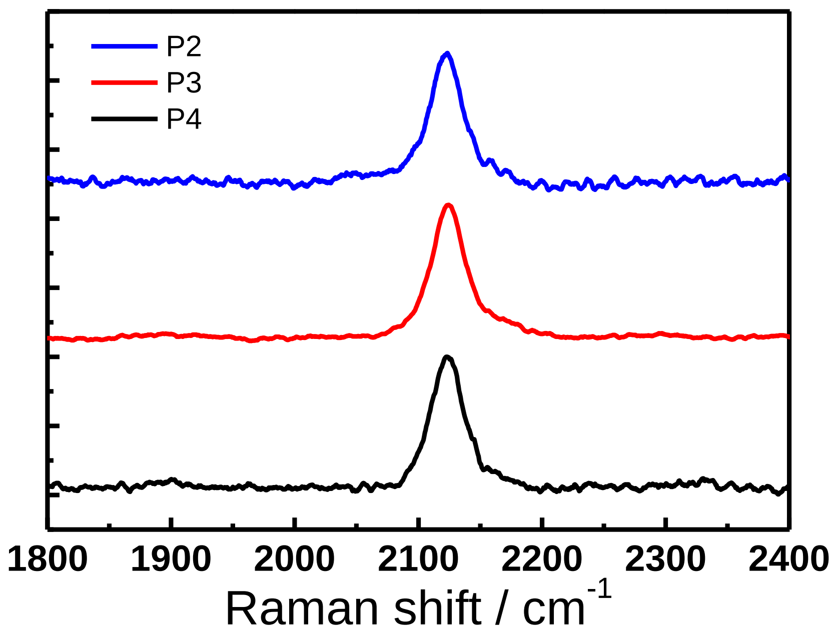

Supplementary Figure 12. Raman spectra of the targeting group functionalized PDDA derivatives. (λ_{Ex} = 488 nm, 0.5 mW)

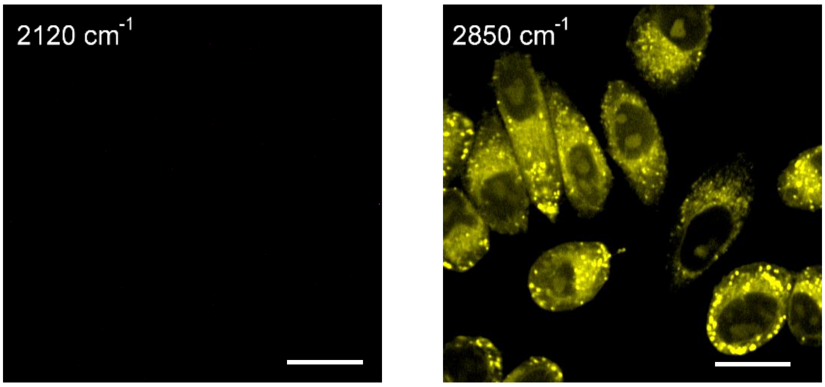

Supplementary Figure 13. SRS images of HeLa cells treated with 50 μM of unmodified PDDA for 48 h. Scale bar: 20 μm.

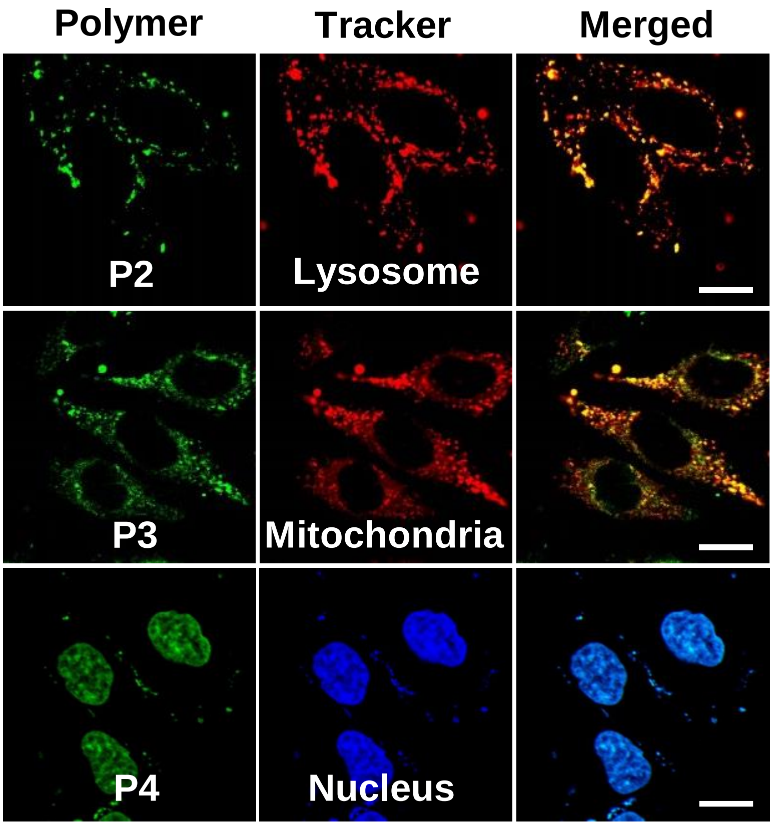

Supplementary Figure 14. CSLM images of HeLa cells treated with PDDA derivatives and co-stained with LysoTracker Red, MitoTracker Deep Red, and DAPI. Green channel for PDDA (λ_{ex} = 488 nm, λ_{em} = 500-550 nm), Red channel for LysoTracker, MitoTracker Deep Red, (λ_{ex} = 640 nm, λ_{em} = 650-750 nm). Blue channel for DAPI (λ_{ex} = 405 nm, λ_{em} = 420-470 nm). Scale bar: 10 μm.

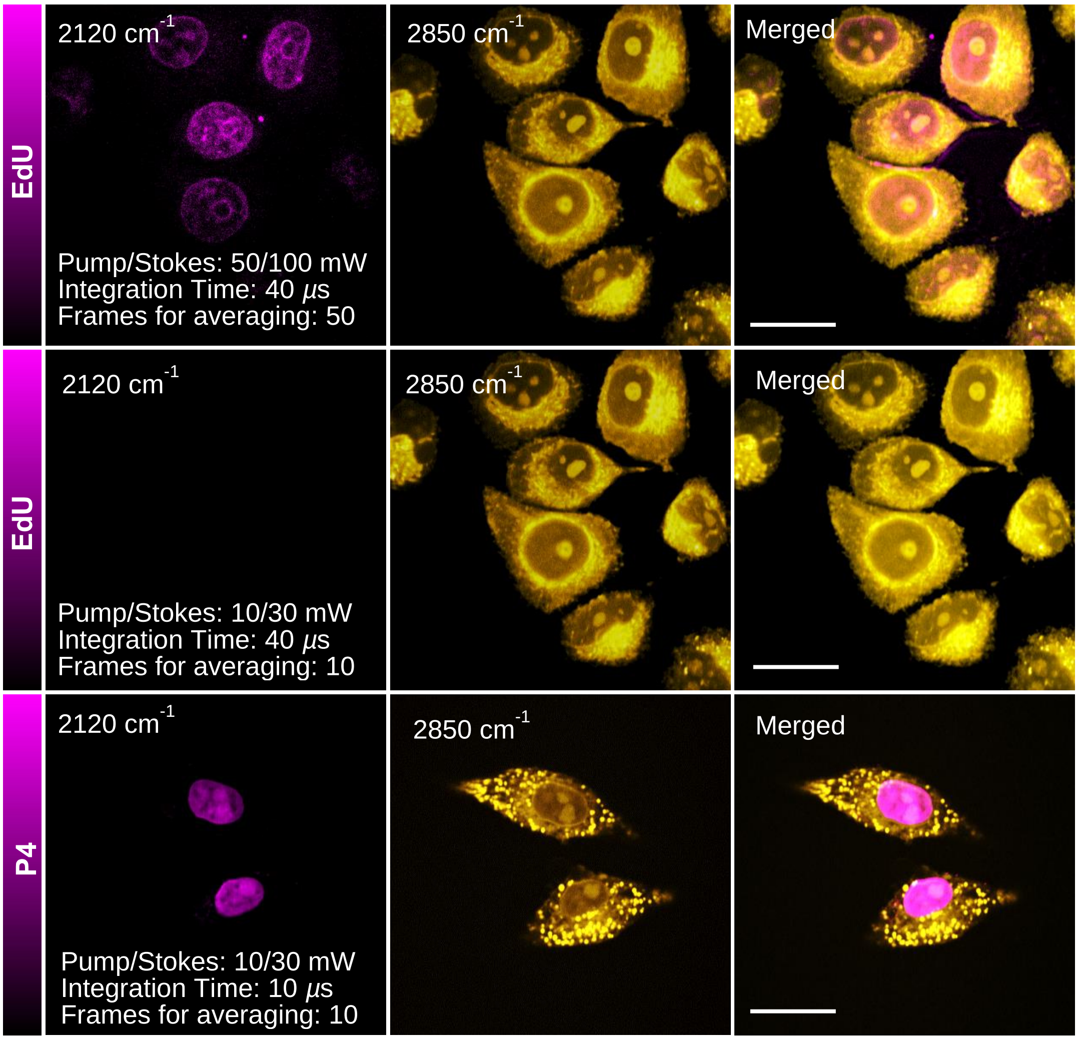

Supplementary Figure 15. SRS images of cells stained by **P4** (50 μM) or EdU (200 μM) at different imaging conditions. Scale bar: 25 μm.

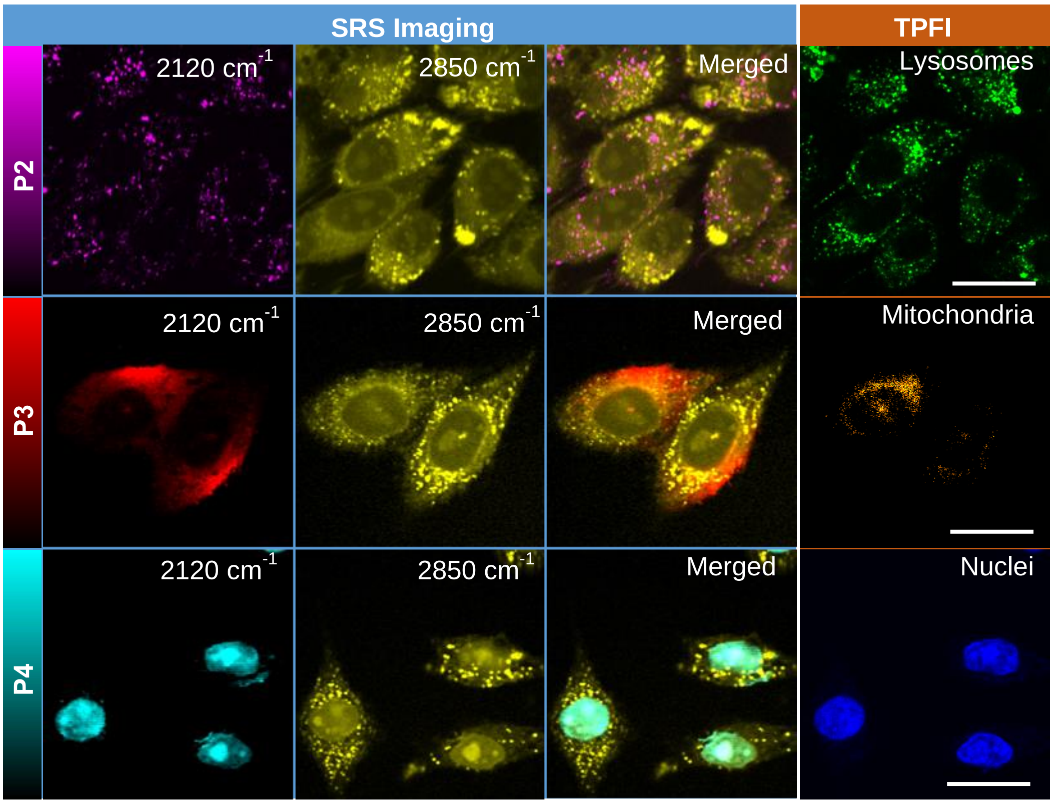

Supplementary Figure 16. SRS images and two-photon fluorescence images (TPFI) of HeLa cells treated with 50 μM of **P2**, **P3**, and **P4** and corresponding fluorescence organelle trackers (lysotracker red, mitotracker green, and DAPI). Scale bar: 25 μm. The mitotracker green is very weak in two-photon excitation, so that its TFPI image is seriously interfered by the endogenous background.

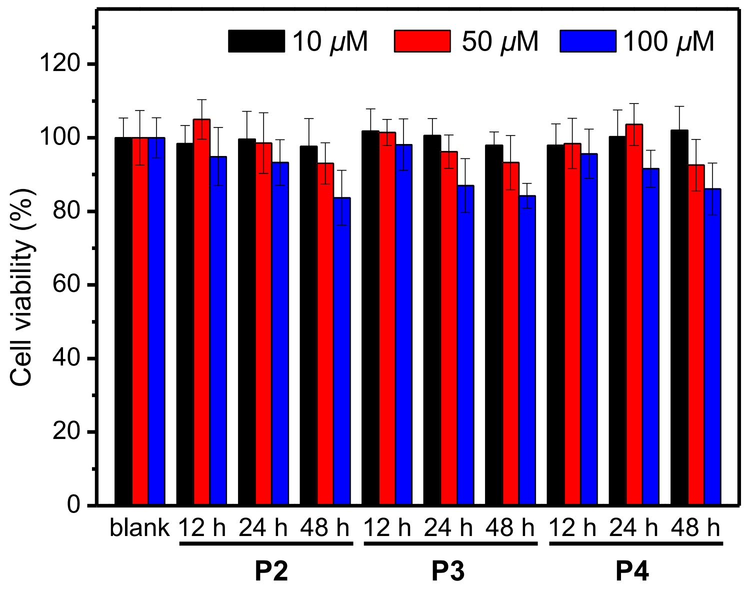

Supplementary Figure 17. MTT assays of PDDA derivatives **P2**, **P3**, and **P4** with different concentrations and incubation time. Data is represented as mean ± standard deviation (n=6).

M+Na+   M-H 2M-H

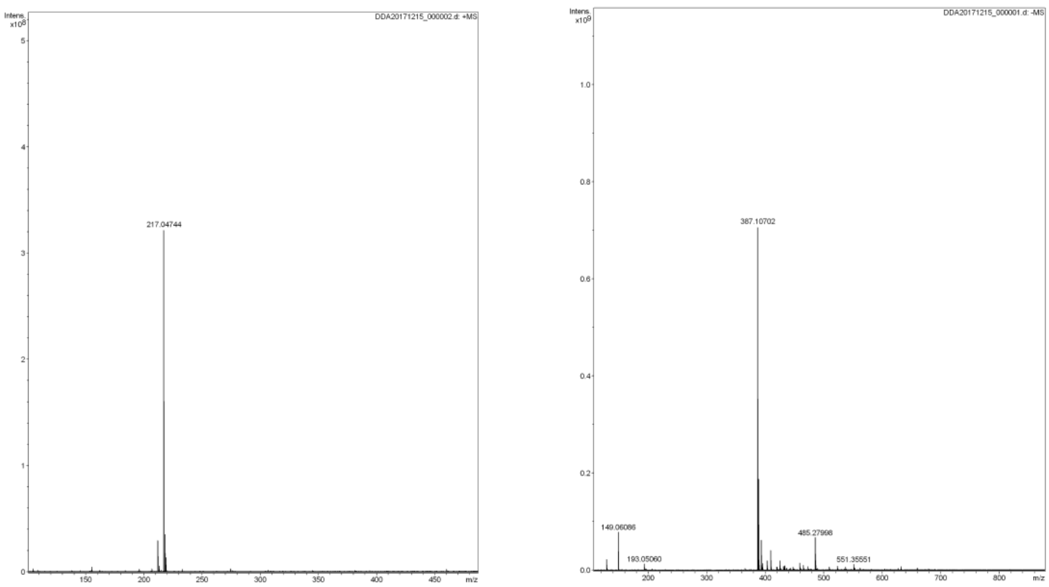

Supplementary Figure 18. HR-MS spectra of the DDA monomer

Supplementary Reference

1. Xu, R., Schweizer, W. B. & Frauenrath, H. Soluble poly (diacetylene) s using the perfluorophenyl− phenyl motif as a supramolecular synthon. *J. Am. Chem. Soc.* **130**, 11437-11445 (2008).
2. Se, K., Ohnuma, H. & Kotaka, T. Urethane-substituted polydiacetylenes: structure and electrical properties of poly [4, 6-decadiyne-1, 10-diol bis [(n-butoxycarbonyl) methyl] urethane]. *Macromolecules* **16**, 1581-1587 (1983).
3. Nakanishi, H. & Kasai, H. Polydiacetylene microcrystals for third-order nonlinear optics. In *ACS Symposium Series*; American Chemical Society **672**, 183-198, (1997).
4. Lim, K., Kapitulnik, A., Zacher, R. & Heeger, A. Conformation of polydiacetylene macromolecules in solution: field induced birefringence and rotational diffusion constant. *J. Chem. Phys.* **82**, 516-521 (1985).
5. Yamakoshi, H., Dodo, K., Palonpon, A., Ando, J., Fujita, K., Kawata, S. & Sodeoka, M. Alkyne-Tag Raman Imaging for Visualization of Mobile Small Molecules in Live Cells. *J. Am. Chem. Soc.* **134**, 20681-20689 (2012).
6. Li, S., Chen, T., Wang, Y., Liu, L., Lv, F., Li, Z., Huang, Y., Schanze, K. S. & Wang, S. Conjugated Polymer with Intrinsic Alkyne Units for Synergistically Enhanced Raman Imaging in Living Cells. *Angew. Chem. Int. Ed.* **56**, 13455-13458 (2017).
7. Hu, F., Zeng, C., Long, R., Miao, Y., Wei, L., Xu, Q. & Min, W. Supermultiplexed optical imaging and barcoding with engineered polyynes. *Nat. Methods* **15**, 194-200 (2018).
